# SCARCE: aSymptomatic respiratory viral Carriage in pre-lung transplant recipients and the effect on eArly-post lung tRansplant CoursE

**DOI:** 10.1016/j.jhlto.2025.100452

**Published:** 2025-11-27

**Authors:** Myrthe B. Bolt, Johanna P. van Gemert, Coretta van Leer-Buter, Erik A.M. Verschuuren, On behalf of TransplantLines, UMCG

**Affiliations:** a Department of Pulmonary Diseases and Tuberculosis, University Medical Centre Groningen, University of Groningen, Groningen, Netherlands; b Department of Medical Microbiology, University of Groningen, University Medical Center Groningen, Groningen, Netherlands

**Keywords:** Lung transplantation, CARV, Asymptomatic viral carriage, Primary graft disfunction

## Abstract

Asymptomatic community-acquired respiratory viral (CARV) carriage before lung transplantation (LTx) has not been studied. This study analyzed CARV carriage pre-LTx and its impact on early post-LTx outcome. This retrospective cohort study included adult LTx recipients at UMC Groningen from January 2017 to August 2023. Pre-LTx viral swabs were routinely tested for CARV. The primary outcome was incidence of primary graft dysfunction (PGD) at 48 or 72 h post-LTx. Secondary outcomes included mechanical ventilation duration, ICU stay, hospital length of stay (LOS), 30- and 90-day mortality, and early rejection therapy. Of 222 recipients, 23 (10.4%) tested positive for a CARV, most commonly rhinovirus (n=10). PGD grade 3 occurred in 14.3% of CARV-positive vs. 9.1% of CARV-negative recipients (p=0.434). No differences were observed in secondary outcomes. Our study provides early data suggesting that asymptomatic carriage may not be associated with worse short-term outcomes.

## Introduction

Asymptomatic community-acquired respiratory viruses (CARV) carriage is quite common^1^, ^2^, ^3^ and might pose a risk to lung transplant (LTx) recipients in the early phase after lung transplantation due to high immunosuppression.^2^ CARV has been associated with chronic lung allograft dysfunction (CLAD),^4^, ^5^ however CARV at the time of LTx remain unexamined.

Primary graft dysfunction (PGD) occurs in up to 30% of cases within the first 3 days post-transplant, causing significant short- and long-term morbidity and mortality.^6^, ^7^ Furthermore, studies have suggested the role of a pro-inflammatory state in PGD development.^6^, ^7^ Yet, the relationship between PGD and asymptomatic CARV carriage at time of LTx, which might also cause an inflammatory state,^8^ remains unexplored. We studied if asymptomatic virus carriage leads to clinical disease during early immunosuppression.

## Materials and methods

### Population

This retrospective cohort study included all adult lung transplant recipients at the University Medical Center Groningen (UMCG), Netherlands, from January 2017 to August 2023. Since 2017, pre-LTx CARV swabs were routinely performed upon arrival for LTx and results usually became available post LTx. Data were collected from the UMCG LTx database and Electronic Patient Records. Recipients without CARV swab data were excluded. Included recipients were asymptomatic or previously infected without signs of active infection. At our center, a transplant team member contacts the patient at the time of a donor offer. If the patient shows symptoms of an exacerbation or infection, they are not admitted for transplantation. No antiviral treatment was given post-LTx in case of a positive swab. Immunosuppression included induction with Basiliximab, followed by Tacrolimus, Mycophenolate mofetil (MMF) and methylprednisolone. Immunosuppression was not modified based on CARV results.

Recipients provided written informed consent, and the study was approved by the UMCG Ethics Committee (CTc 18250).

Primary outcome was the incidence of PGD stage 3 at 48 h or 72 h post-LTx, categorized according to the International Society for Heart and Lung Transplantation (ISHLT) classification.^6^ Secondary outcomes included duration of post-LTX mechanical ventilation, intensive care unit (ICU) stay, hospital length of stay (LOS), 30-day and 90-day mortality rate, and incidence of acute rejection within 90 days post-LTx. During the first three months post-transplant, rejection is often diagnosed clinically. Therefore, data on specific type of rejection is not routinely available. Rejection therapy consisted of pulse methylprednisolone.

### Statistical analysis

For quantitative outcomes, median and interquartile range (IQR) were used by swab result. Group differences were tested with the Mann-Whitney U test. For qualitative outcomes, frequencies and percentages were compared using Fisher’s exact or Chi-square test. A two-tailed p-value <0.05 was considered significant. Analyses were performed in SPSS23. Recipients who died within 72 h post-LTx were excluded from all outcomes except mortality. Logistic regression was performed to identify possible confounders.

### Virology

Positive CARV was defined as positive polymerase chain reaction (PCR) of a respiratory sample for rhinovirus, respiratory syncytial virus (RSV), human metapneumovirus (hMPV), influenza virus, para-influenza virus (PIV), and coronaviruses including SARS-CoV-2 (COVID-19). Samples included nasopharyngeal swabs, nasal washes and sputum. PCR was tested as described before.^9^

## Results

**Figure 1:**
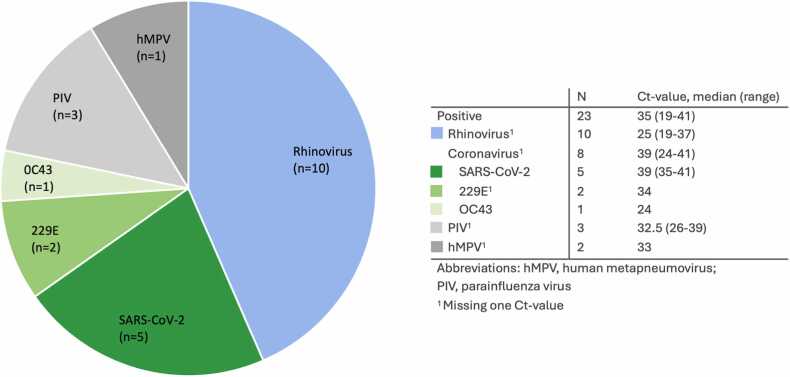
CARV swab results.

Of 243 recipients transplanted during the study period, 21 were excluded due to missing data. Of the 222 recipients, 23 (10.4%) had a positive pre-LTx CARV swab (Table 1 and Figure 1). Detected viruses included Rhinovirus (n=10), SARS-CoV-2 (n=5), Coronaviruses OC43/229E (n=3), PIV (n=3), and hMPV (n=2) (Figure 1). The median time from positive test until LTx was 10.9 h (IQR 4.7–14.8 h).

**Table 1:** Recipient Demographics

	All Recipients	Pre-LTx CARV Swab		
	Total	Positive	Negative	p-value
Recipients	222	23	199	
Sex, female (%)	105 (47.3)	7 (30.4)	98 (49.2)	0.087^b^
Age, y (IQR)	57.8 (50.1-62.2)	58.0 (51.2-60.9)	58.6 (51.9-62.7)	0.424^c^
Underlying disease, n (%)				0.747^d^
Emphysema/ COPD	116 (52.4)	11 (47.8)	105 (52.8)	
Cystic fibrosis	9 (4.1)	0 (0.0)	9 (4.5)	
Pulmonary hypertension	25 (11.3)	2 (8.7)	23 (11.6)	
Fibrosis	56 (25.2)	8 (34.8)	48 (24.1)	
Graft-failure	5 (2.3)	1 (4.3)	4 (2.0)	
Bronchiectasis	3 (1.4)	0 (0.0)	3 (1.5)	
Other^a^	8 (3.6)	1 (4.3)	7 (3.5)	
Transplant type, n (%)				1.00^d^
Single	9 (4.1)	1 (4.3)	8 (4.0)	
Double	212 (95.5)	22 (95.7)	190 (95.5)	
Lung-liver	1 (0.5)	0 (0.0)	1 (0.5)	

Continuous data are displayed as medians with interquartile range (IQR)

Abbreviations: COPD, chronic obstructive pulmonary disease.

a Other underlying disease (histiocytosis, sarcoidosis, Stevens-Johnson syndrome, graft versus host disease, bronchiectasis, post-SARS-cov-2)

b *P*-value from Chi-square test

c *P*-value from Mann-Whitney U test

d *P*-value from Fisher’s exact

Overall, 9.6% (19/197) of recipients developed PGD grade 3 at 48 or 72 h. PGD grade 3 occurred in 14.3% (3/21) of CARV-positive and 9.1% (16/174) of CARV-negative recipients (p=0.434) (Table 2). There were no significant differences in duration of post transplant mechanical ventilation, ICU stay, total hospital LOS, 30-day mortality rate, occurrence of acute rejection (Table 2). Focusing on SARS-CoV-2, we saw a longer ventilation time, but no excess mortality. (Table 3). Two SARS-CoV-2–related cases were transplanted for COVID-induced lung fibrosis and were bridged with invasive ventilation.

**Table 2:** Post-LTx Outcomes

	All Recipients	Pre-LTx CARV Swab		
	Total	Positive	Negative	p-value
Recipients	222	23	199	
PGD at 48 or 72 h n, (%)				0.434^a^
Grade 0-2 Grade 3	178 (90.4) 19 (9.6)	18 (85.7) 3 (14.3)	160 (90.9) 16 (9.1)	
Duration mechanical ventilation, days (IQR)	1 (1-5)	2 (1-13)	1 (1-4)	0.318^b^
ICU stay, days (IQR)	5 (3-13)	12 (2-26)	5 (3-11)	0.434^b^
Hospital LOS, days (IQR)	33 (23-50)	37 (29-65)	32 (23-49)	0.216^b^
Rejection therapy use^d^^,^ n (%)	91 (41.0)	8 (34.8)	83 (41.7)	0.656^c^
30-day mortality, n (%)	11 (5.0)	0 (0.0)	11 (5.5)	0.610^a^
90-day mortality, n (%)	20 (9.0)	0 (0.0)	20 (10.1)	0.238^a^

Continuous data are displayed as medians with interquartile range (IQR)

Abbreviations: ICU, intensive care unit; LOS, length of stay; PGD, primary graft dysfunction.

a P-value from Fisher’s exact

b *P*-value from Mann-Whitney U test

c *P*-value from Chi-square test

d Rejection therapy included methylprednisolone.

**Table 3:** Post-hoc Analysis of COVID-19 and Post-LTx Outcomes

	All Recipients	Pre-LTx CARV Swab		
	Total	COVID-19	Negative	p-value
Recipients	204	5	199	
PGD at 48 or 72 h n, (%)				0.393^1^
Grade 0-2 Grade 3	164 (90.6) 17 (9.4)	4 (80.0) 1 (20.0)	160 (90.9) 16 (9.1)	
Duration mechanical ventilation, days (IQR)	1 (1-5)	8 (5.5-14)	1 (1-4)	0.008^2^
ICU stay, days (IQR)	5 (3-12.5)	15 (11-33.5)	5 (3-11)	0.022^2^
Hospital LOS, days (IQR)	32 (23-48.5)	42 (25-61.5)	32 (23-49)	0.620^2^
Rejection therapy use3, n (%)	85 (41.7)	2 (40.0)	83 (41.7)	1.00^1^
30-day mortality, n (%)	11 (5.0)	0 (0.0)	11 (5.5)	1.00^1^
90-day mortality, n (%)	20 (9.0)	0 (0.0)	20 (10.1)	1.00^1^

Continuous data are displayed as medians with interquartile range (IQR)

Abbreviations: ICU, intensive care unit; LOS, length of stay; PGD, primary graft dysfunction.

1 P-value from Fisher’s exact

2 *P*-value from Mann-Whitney U test

3 Rejection therapy included methylprednisolone.

In multivariate analysis, male sex was associated with lower risk for PGD (OR 0.25, 95% CI 0.08–0.72, p=0.011), whereas CARV positivity showed no higher risk (OR 2.08, 95% CI 0.52–8.24, p=0.298) (Table 4).

**Table 4:** Univariate and Multivariate Regression Analysis

	Univariate OR (95% CI)	p-value	Multivariate OR (95% CI)	p-value
Sex	0.26 (0.09–0.75)	0.013	0.25 (0.08–0.72)	0.011
Age	0.98 (0.94–1.02)	0.263	-	-
Underlying disease	1.19 (0.92–1.53)	0.186	-	-
Transplant type^1^ (double vs other)	1.01 (0.05–19.8)	0.998	-	-
CARV positive	1.67 (0.44-6.28)	0.450	2.08 (0.52-8.24)	0.298

Abbreviations: OR, odds ratio; CI, confidence interval

1 Due to small numbers in this category, analysis was performed using ‘Double’ versus ‘Other’ transplants.

## Discussion

This study shows that pre-LTx CARV carriage is common (10.4%) among asymptomatic LTx candidates. Our findings suggest that asymptomatic CARV carriage does not impact early outcomes, however the limited sample size restricts firm conclusions.

Our CARV detection rate is slightly higher than rates among ambulatory adults in New York (4.3–6%), which is likely due to increased susceptibility in pre-LTx recipients with underlying lung disease.^10^ Moreover, our study aligns with asymptomatic CARV rates reported among LTx recipients on average 3–4 years after LTx.^3^, ^11^ Rhinovirus was most frequently detected (43%) in our study cohort, consistent with prior studies.^1^, ^3^, ^11^, ^12^

Thus far, studies have shown that symptomatic infections are linked to adverse outcomes such as CLAD or graft loss, while asymptomatic infections do not.^3^, ^5^ Our findings are consistent with this and expand on the early post-LTx period. Similarly, a study analyzing CARV in bronchoscopy samples one day post-LTx found no association with PGD.^12^ This likely reflects donor-derived rather than recipient-derived CARV, given the lower respiratory tract sampling. In contrast, our study focused exclusively on recipient-derived CARV.

In our study, all of our recipients were asymptomatic upon arrival for LTx despite a positive swab. This may be because respiratory symptoms may be masked in patients with end-stage lung disease. Alternatively, viral RNA can be obtained at any point during infection, encompassing recipients recovering from a CARV, who continue to shed the virus post-infection, a phenomenon well-documented in COVID-19 cases.^8^ This could explain the high Ct values, reflecting minimal viral loads, however some recipients had higher loads.

Among the five SARS-CoV-2–positive recipients, two were transplanted due to COVID-induced lung fibroses and were bridged with invasive ventilation pre-transplant, possibly contributing to longer post-transplantation ventilation. Post hoc analysis showed no increase in mortality or hospital LOS.

This study’s retrospective design and small sample size are important limitations. We could not stratify outcomes by virus type or viral load, and seasonal trends were not assessed. The broad time frame (2017–2023) spans the COVID-19 pandemic, when respiratory virus circulation patterns were disrupted. Non-COVID CARVs were virtually undetected early in the pandemic, likely underestimating asymptomatic CARV carriage among our cohort. Furthermore, follow-up swabs were not routinely obtained post-LTx, limiting our understanding of viral clearance or recurrences. However, there was no clinical suspicion of CARV post-LTx in these recipients.

Although treating detected CARV peri-transplant may seem intuitive, treatment options are limited for most CARV. Our study aimed to assess whether these CARV posed an unrecognized clinical issue rather than to guide antiviral therapy, making this an initial exploration of their peri-transplant relevance.

In conclusion, this study provides early data on pre-LTx asymptomatic CARV carriage, suggesting asymptomatic carriage is not associated with worse short-term outcomes. Importantly, our results show no reason to cancel a LTx due to asymptomatic CARV positivity. Overall, we believe routine testing of asymptomatic LTx candidates for CARV offers limited added value.

## Funding

This research did not receive any specific grant from funding agencies in the public, commercial, or not-for-profit sectors.

## Declaration of Generative AI and AI-assisted technologies in the writing process

During the preparation of this work the author(s) used ChatGPT during the writing process in order to improve the readability and language of the manuscript. After using this tool/service, the author(s) reviewed and edited the content as needed and take(s) full responsibility for the content of the published article.

## Declaration of Competing Interest

The authors declare that they have no known competing financial interests or personal relationships that could have appeared to influence the work reported in this paper.

## Data Availability

Data not publicly available.
